# Adjuvant FLOT provides survival benefit for oesophagogastric junction and gastric adenocarcinoma patients with low tumour regression after neoadjuvant chemotherapy

**DOI:** 10.1002/ijc.70048

**Published:** 2025-07-22

**Authors:** Max Kraemer, Naita M. Wirsik, Hakan Alakus, Hans A. Schloesser, Hans Fuchs, Wolfgang Schroeder, Christiane J. Bruns, Su Ir Lyu, Friederike Baehr, Thomas Zander, Alexander Quaas

**Affiliations:** ^1^ Department I of Internal Medicine, Center for Integrated Oncology Aachen Bonn Cologne Duesseldorf, Gastrointestinal Cancer Group Cologne GCGC University of Cologne Cologne Germany; ^2^ Department of General, Visceral, Cancer and Transplantation Surgery University Hospital Cologne Cologne Germany; ^3^ Faculty of Medicine and University Hospital of Cologne, Institute of Pathology University of Cologne Cologne Germany

**Keywords:** adjuvant chemotherapy, FLOT, gastric adenocarcinoma, oesophagogastric junction adenocarcinoma, tumour regression

## Abstract

Oesophagogastric junction and gastric adenocarcinoma (OGA) are associated with high mortality rates, with 5‐year survival rates below 50% in the curative setting. This study evaluates the efficacy of adjuvant chemotherapy (a chemotherapy regimen consisting of docetaxel, oxaliplatin, leucovorin and 5‐fluorouracil [FLOT]) in patients with low tumour regression grades (TRG) following neoadjuvant FLOT (>10% viable tumour cells in surgical specimen, TRG 2/3 analogue Becker's classification). Data from all patients who had undergone ≥3 cycles of neoadjuvant FLOT with R0 resection and TRG 2/3 in surgical specimen, diagnosed between 2017 and 2020 at the University of Cologne (*n* = 134), were analyzed. Patients were categorised into three groups based on the administration of postoperative FLOT: ‘FLOT complete’ (four cycles), ‘FLOT incomplete’ (one to three cycles) and ‘no FLOT’ (0 cycles). Progression‐free survival (PFS) and overall survival (OS) were compared. There is a statistically significant PFS advantage for the ‘FLOT complete’ group compared to ‘no FLOT’ (*p* = .028) in the total patient cohort and a tendency for an OS benefit. In the subgroup of patients with lymph node metastasis in surgical specimen (ypN+ cohort, *n* = 91), the PFS advantage of ‘FLOT complete’ was diminished and statistically no longer significant, and there is no OS benefit for these patients. However, multivariate analysis confirmed a significant PFS benefit for ‘FLOT complete’ both in the total cohort (*p* = .011) and in ypN+ patients (*p* = .018). These findings suggest that full adjuvant FLOT is beneficial even for OGA patients with low tumour regression; however, its efficacy appears reduced in those with lymph node metastasis, warranting further investigation into individualising treatment strategies.

## What's New?

Perioperative FLOT, a chemotherapy regimen consisting of docetaxel, oxaliplatin, leucovorin and 5‐fluorouracil, is the standard treatment for oesophagogastric junction and gastric adenocarcinoma (OGA). Whether patients benefit further from adjuvant FLOT, given postoperatively, is uncertain. This study assessed adjuvant FLOT in a large cohort of OGA patients with low tumour regression (TRG2/3) following surgery. Significant gains in survival were observed in patients who received both neoadjuvant and postoperative FLOT. This benefit was diminished in lymph node‐positive patients. The findings indicate that adjuvant FLOT is beneficial, but only for some patients, highlighting a need for personalised treatment strategies in OGA.

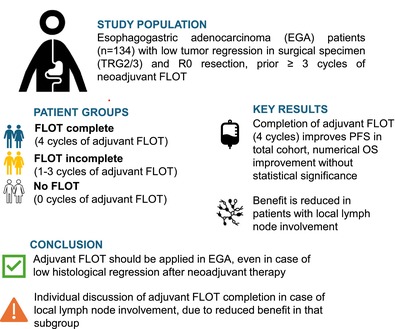

**Abbreviations**
CI: confidence interval
CROSS: a specific chemoradiotherapy regimen for oesophageal cancer
ctDNA: circulating tumour DNA
ESMO: European Society for Medical Oncology
FLOT: a chemotherapy regimen consisting of docetaxel, oxaliplatin, leucovorin and 5‐fluorouracil
HR: hazard ratio
IPTW: inverse probability of treatment weighting
MSI‐H: microsatellite instability‐high (a subtype of cancer)
OGA: oesophagogastric junction and gastric adenocarcinoma
OS: overall survival
pCR: pathological complete response
PFS: progression‐free survival
TRG: tumour regression grade
ypN: pathologic nodal stage after neoadjuvant therapy
ypT: pathologic tumour stage after neoadjuvant therapy

## 1 INTRODUCTION

Oesophagogastric junction and gastric adenocarcinoma (OGA) are among the cancer entities with the highest mortality rates, and even in a curative setting, the 5‐year survival rate is less than 50%.^1^, ^2^, ^3^ In locally advanced tumour stages without distant metastasis, multimodal treatment approaches are often indicated. The current European Society for Medical Oncology (ESMO) guidelines recommend perioperative chemotherapy according to the FLOT regimen for gastric adenocarcinomas starting from Union for International Cancer Control (UICC) stage IB (>T1 and/or ≥N0, M0), while for locally advanced adenocarcinomas of the oesophagogastric junction, both chemotherapy regimen consisting of docetaxel, oxaliplatin, leucovorin and 5‐fluorouracil (FLOT) and neoadjuvant chemoradiotherapy according to the specific chemoradiotherapy regimen for oesophageal cancer (CROSS) protocol are approved therapy concepts.^4^, ^5^ In this context, the recently published first results of the ESOPEC trial demonstrated a superiority of perioperative therapy with FLOT over the CROSS protocol for oesophageal adenocarcinoma, with approximately 7% higher 3‐year survival.^6^ In contrast, the addition of preoperative radiochemotherapy to conventional perioperative chemotherapy for OGA patients did not result in improved progression‐free survival (PFS) or overall survival (OS) in the recently published results of the TOPGEAR study.^7^ Currently, the addition of immune checkpoint inhibition to perioperative treatment is further being investigated in the MATTERHORN, KEYNOTE‐585 and DANTE trials.^8^, ^9^, ^10^ While increased pathological complete response (pCR) rates have already been observed with the addition of checkpoint blockade, the demonstration of a survival benefit in the future could potentially lead to a modification of the current perioperative FLOT standard. In the adjuvant setting, the EORTC VESTIGE trial recently demonstrated that postoperative immunotherapy alone (ipilimumab/nivolumab) was inferior to adjuvant chemotherapy in high‐risk OGA patients (R1 resection and/or ypN+), confirming that the continuation of postoperative FLOT remains the current standard of care for this patient population.^11^

The FLOT‐protocol consists of four neoadjuvant cycles of docetaxel 50 mg/m^2^, oxaliplatin 85 mg/m^2^, leucovorin 200 mg/m^2^ and 5‐Fluorouracil (5‐FU) 2600 mg/m^2^ on Day 1, every 2 weeks, followed by surgical resection, typically performed approximately 4–6 weeks after the last dose of neoadjuvant chemotherapy. Postoperatively, the treatment regularly continues with four additional cycles of FLOT.^3^ However, the FLOT‐4‐study by Al‐Batran et al. showed that only 213 of the 301 (70.8%) R0‐resected patients started the adjuvant part of the therapy.^3^ In their study, lack of efficacy/disease progression, patients' requests or unacceptable toxicity were the most common reasons for discontinuation. Also in clinical practice, the question often arises about which patient groups actually benefit from adjuvant FLOT therapy, given the significant side effects of the treatment (e.g., 71% experiencing polyneuropathy of any grade and 51% grade 3/4 neutropenia).^3^ In particular, patients with a high proportion of viable tumour cells in the surgical specimen and therefore low tumour regression grade (TRG) are the focus of attention. Different grading systems exist for classifying tumour regression in OGA, while the classification according to Becker et al. is often used in Europe^12^: here, a (sub‐)total tumour regression (TRG 1a/b) is assumed with <10% viable tumour cells in the surgical specimen, while ≥10% viable tumour cells correspond to a partial (TRG2) or minor (>50%; TRG3) response. This distinction has drastic consequences for the prognosis of the patients, with a 3‐year disease‐free survival of 74.9% at TRG 1a/b compared to 43.5% at TRG 2 and 3.^13^ In terms of continuing adjuvant FLOT‐chemotherapy guided by tumour regression in OGA, two lines of reasoning may logically emerge: 1. Patients with low tumour regression are high‐risk patients and should receive the maximum amount of cytotoxic therapy; thus, the adjuvant therapy should continue with four cycles of FLOT. 2. Patients with low tumour regression gain very little benefit from the therapy, suggesting that, in favor of reducing side effects and improving quality of life, the adjuvant continuation with FLOT should be avoided or at least reduced.

To date, only very few studies support clinical decision‐making in this setting. Among them, the recently published SPACE‐FLOT study is particularly relevant.^14^ In this multicentre analysis of 1.887 patients treated with perioperative FLOT, adjuvant therapy showed no survival benefit in patients with pCR or minimal/no response, but improved outcomes in those with partial response. It should be noted, however, that the definition of ‘partial response’ in this study does not correspond to that of the Becker classification (TRG2). Instead, it broadly includes patients who had neither a complete response (0% viable tumour cells) nor no response (100% viable tumour cells), as outlined in Table S2 of their publication. Although conclusions regarding the benefit of adjuvant FLOT in relation to the extent of viable tumour cells cannot be drawn from this analysis, the study nonetheless provides important first evidence that continuation of FLOT therapy may be beneficial even in patients with limited histopathological tumour regression. To date, no other large‐scale studies are available that confirm or refute these findings using a consistent tumour regression grading system. As a major European centre for gastric and oesophageal surgery, we have therefore evaluated all OGA patients with an initial diagnosis between 2017 and 2020 and TRG 2/3 analogue to the Becker's classification in the surgical specimen, and we analyzed their follow‐up data. We compare the completion of a full adjuvant treatment with four cycles of FLOT (‘FLOT complete’) with incomplete/terminated adjuvant FLOT (‘FLOT incomplete,’ one to three cycles) and no adjuvant therapy (0 cycles, ‘no FLOT’), regarding survival data for each approach.

## 2 MATERIALS AND METHODS

### 2.1 Study design

**FIGURE 1:**
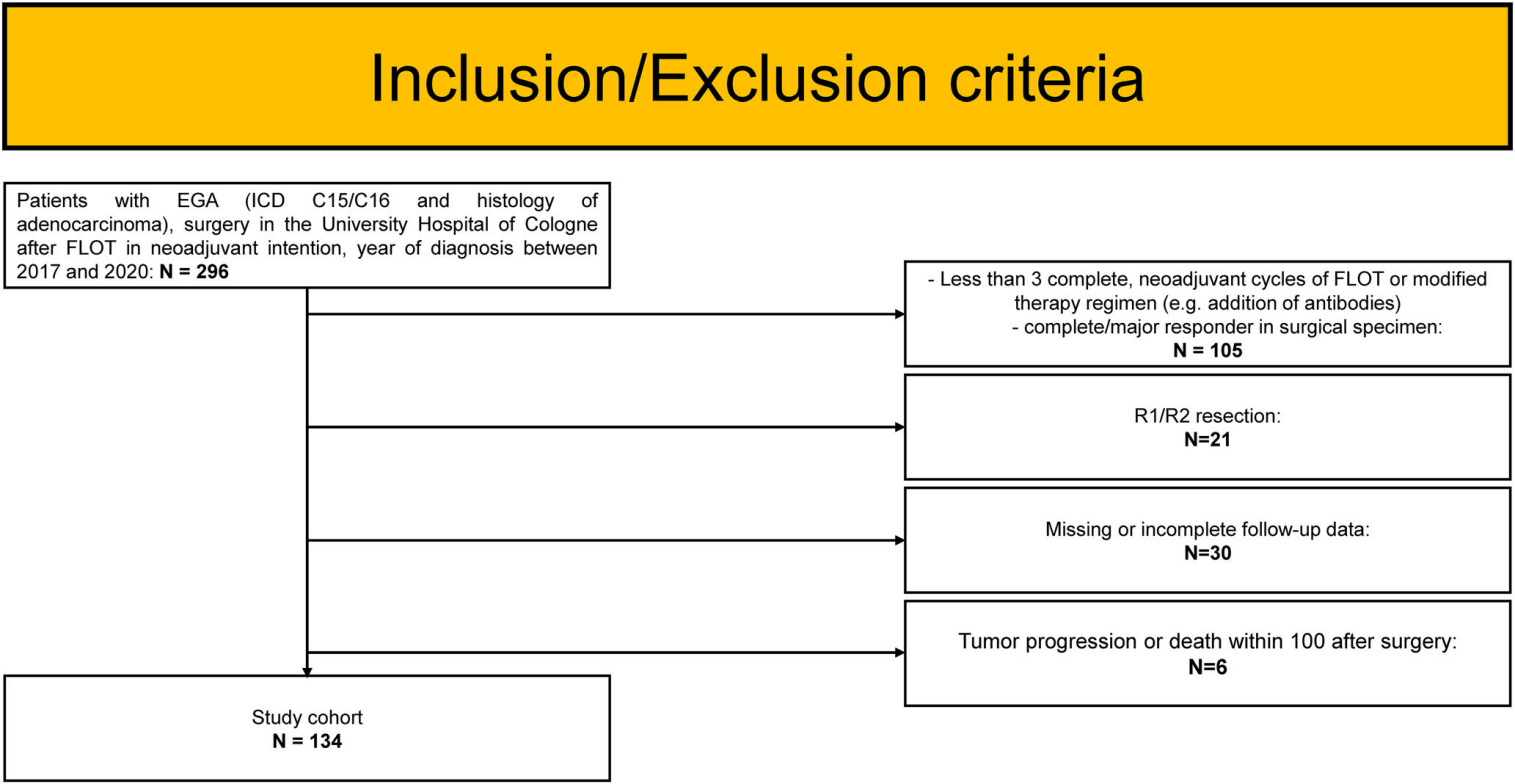
Inclusion/exclusion criteria—flowchart. OGA, oesophagogastric junction and gastric adenocarcinoma; FLOT, a chemotherapy regimen consisting of docetaxel, oxaliplatin, leucovorin and 5‐fluorouracil.

To identify patients with OGA and low tumour regression, we systematically analyzed our internal clinical databases and identified 296 patients with an initial diagnosis between 1 January 2017 and 31 December 2020, who underwent surgical resection of their primary tumour at the Department of General, Visceral and Cancer Surgery, University of Cologne, Germany, after neoadjuvant treatment according to the FLOT regimen. We then excluded patients who received less than three full cycles of neoadjuvant FLOT, a modified regimen (e.g., addition of antibodies as part of a study), or had less than 10% viable tumour cells in the surgical specimen (*N* = 105). Of the remaining 191 patients with TRG 2/3 after FLOT, we analyzed only those with R0 resection (*n* = 170). The patients' follow‐up data were collected within a structured follow‐up programme extending up to 5 years after surgery. Missing information was supplemented in collaboration with outpatient clinics. Follow‐up data could still not be generated for 30 patients (17.6%). We also excluded patients with disease progression or death within 100 days post‐surgery (*N* = 6) to ensure that (a) patients with early progression—possibly already present at the time of surgery—were not included, as no meaningful adjuvant effect of FLOT could be expected or evaluated in such cases, and (b) surgery‐related deaths were excluded from the analysis. This resulted in 134 patients with complete datasets included in our analysis (compare Figure 1). We selected PFS as the primary endpoint, as it primarily reflects the effect of adjuvant FLOT therapy, and OS as the secondary endpoint, since it may also be influenced by subsequent lines of treatment.

### 2.2 Statistics

The structural equality among three groups was evaluated using multiple contingency tables, analyzed with the chi‐square test of independence. For each contingency table, the distribution of categorical variables across the groups was assessed to determine if significant differences existed. The chi‐square test was employed to compare the observed frequencies with expected frequencies under the null hypothesis of no association. When expected cell counts were below 5, the Monte Carlo simulation was applied to ensure the accuracy of the *p*‐values. In addition to the group comparisons, univariate analyses were performed to assess the association between individual clinical variables and survival outcomes prior to multivariate adjustment. Kaplan–Meier curves were generated to compare survival across three groups, with pairwise differences assessed using the Log‐Rank test. A multivariate Cox proportional hazards regression was conducted to evaluate whether the independent variables significantly influenced progression‐free (PFS) and OS, assessing their impact while controlling for other covariates. An inverse probability of treatment weighting (IPTW) analysis was further performed to control for confounding. Covariates included age, sex, tumour localisation, histology, signet ring cells, ypT, ypN and surgical approach. Propensity scores were estimated via multinomial logistic regression, and IPTWs were assigned accordingly. Cases with extreme weights (>10) were excluded. Weighted Kaplan–Meier analysis was conducted accordingly. For statistical analysis, SPSS Statistics for Windows (version 29) was used.

## 3 RESULTS

### 3.1 Patients' characteristics

Patients' characteristics are given in Table 1. The median age was 61 years (range 23–82 years), with patients who completed all four cycles of FLOT having a median age of 59.5 years, while those with incomplete or no adjuvant FLOT therapy had median ages of 63 and 62 years, respectively (*p* = .168). The cohort consisted of 82.8% male and 17.2% female patients with no significant differences between the three subgroups. Median follow‐up time for PFS was 14.39 months (range 3.38–78.52 months); median follow‐up time for OS was 28.15 months (range 4.01–78.52 months).

**TABLE 1:** Baseline characteristics of all included patients.

	All	FLOT complete (4 cycles)	FLOT incomplete (1–3 cycles)	No FLOT (0 cycles)	*p*‐Value
Patients	134	86 (64.2%)	27 (20.1%)	21 (15.7%)	
Age					
Median (range)	61 (23–82)	59.5 (23–77)	63 (28–79)	62 (50–82)	
≤60	65 (48.5%)	47 (54.7%)	10 (37.0%)	8 (38.1%)	.168
>60	69 (51.5%)	39 (45.3%)	17 (67.0%)	13 (61.9%)	
Sex					
Female	23 (17.2%)	18 (20.9%)	3 (11.1%)	2 (9.5%)	.318
Male	111 (82.8%)	68 (79.1%)	24 (88.9%)	19 (90.5%)	
ypT					
ypT1	15 (11.2%)	6 (7.0%)	7 (25.9%)	2 (9.5%)	.033
ypT2	15 (11.2%)	7 (8.1%)	4 (14.8%)	4 (19.9%)	
ypT3	83 (61.9%)	55 (64.0%)	14 (51.9%)	14 (66.7%)	
ypT4	21 (15.7%)	18 (20.9%)	2 (7.4%)	1 (4.8%)	
ypN					
ypN0	43 (32.1%)	29 (33.7%)	10 (37.0%)	4 (19.0%)	.626
ypN1	32 (23.9%)	19 (22.1%)	8 (29.6%)	5 (23.8%)	
ypN2	23 (17.1%)	16 (18.6%)	2 (7.4%)	5 (23.8%)	
ypN3	36 (26.9%)	22 (25.6%)	7 (25.9%)	7 (33.3%)	
Localisation					
AEG I	36 (26.9%)	22 (25.6%)	7 (25.9%)	7 (33.3%)	.321
AEG II	56 (41.8%)	34 (39.5%)	10 (37.0%)	12 (57.1%)	
AEG III	13 (9.7%)	10 (11.6%)	2 (7.4%)	1 (4.8%)	
Stomach	29 (21.6%)	20 (23.3%)	8 (29.6%)	1 (4.8%)	
Eastern Cooperative Oncology Group (ECOG)					
0	56 (41.8%)	40 (29.9%)	11 (40.7%)	5 (23.8%)	.417
1	42 (31.3%)	26 (30.2%)	7 (25.9%)	9 (42.9%)	
2	5 (3.7%)	3 (3.5%)	1 (3.7%)	1 (4.8%)	
n.a.	31 (23.1%)	17 (19.7%)	8 (29.6%)	6 (28.6%)	
Laurens classification					
Intestinal	65 (48.5%)	39 (45.3%)	12 (44.4%)	14 (66.7%)	.481
Diffuse	34 (25.4%)	24 (27.9%)	7 (25.9%)	3 (14.3%)	
Mixed	35 (26.1%)	23 (26.7%)	8 (29.6%)	4 (19.0%)	
Signet ring cells					
No	113 (84.3%)	70 (81.4%)	23 (85.2%)	20 (95.2%)	.291
Yes	21 (15.7%)	16 (18.6%)	4 (14.8%)	1 (4.8%)	
Type of surgery					
Oesophagectomy	90 (67.2%)	54 (62.8%)	17 (63.0%)	19 (90.5%)	.046
Gastrectomy	44 (32.8%)	32 (37.2%)	10 (37.0%)	2 (9.5%)	

*Note*: *p*‐Values are reported for comparison between the three different cohorts ‘FLOT complete,’ ‘FLOT incomplete’ and ‘no FLOT.’

Abbreviations: AEG, adenocarcinoma of the oesophagogastric junction, analogue Siewert‐classification; FLOT, a chemotherapy regimen consisting of docetaxel, oxaliplatin, leucovorin, and 5‐fluorouracil; ypN, pathologic nodal stage after neoadjuvant therapy; ypT, pathologic tumour stage after neoadjuvant therapy.

The distribution of ypT stages showed statistically significant differences, with ypT3 as the most common stage overall (61.9%). Notably, the ‘FLOT incomplete’ group had a slightly higher proportion of less advanced ypT1/2 tumours (40.7%) compared to the ‘FLOT complete’ and ‘no FLOT’ groups, where these stages were less frequent (15.1% and 30.4%, respectively). In contrast, the ‘FLOT complete’ group had the highest proportion of ypT4 tumours (20.9%). In addition, there was a marginally significant difference in the type of surgical procedure, with a higher proportion of oesophagectomies in the ‘no FLOT’ group (90.5%) compared to the other two groups (‘FLOT incomplete’: 63.0%, ‘FLOT complete’: 62.8%). Biomarkers such as HER2 amplification, Programmed Death‐Ligand 1 (PD‐L1) status and microsatellite instability‐high (a subtype of cancer) (MSI‐H) were not routinely tested in the curative setting and are therefore only sparsely available; details are presented in Table S3.

Furthermore, no statistically significant differences were observed regarding the ypN stage, tumour localisation, ECOG performance status, histological tumour type according to Lauren's classification, and proportion of signet ring cells between the three subgroups. Concerning the ypN status, a total of 91 patients (67.9%) showed lymph node metastasis in the surgical specimen, while 43 patients (32.1%) had no lymph node involvement.

### 3.2 Postoperative FLOT‐therapy

In total, 134 patients with at least 10% viable tumour cells in the surgical specimen and R0 resection after neoadjuvant FLOT therapy were included in this study. Of these, 86 patients received all four cycles of adjuvant FLOT (64.2%, ‘FLOT complete’), 27 patients initiated but did not complete adjuvant chemotherapy (one to three cycles; 20.1%, ‘FLOT incomplete’) and 21 patients received no adjuvant chemotherapy (15.7%, ‘no FLOT’). In detail, six patients (22.2%) in the ‘FLOT incomplete’ group received one postoperative cycle of FLOT, seven patients (25.9%) received two cycles and 14 patients (51.9%) received three cycles. The most common reasons for complete omission of adjuvant therapy (‘no FLOT’ group) were poor performance status (42.9%) and delayed postoperative recovery (23.8%). A detailed overview of all reasons is provided in Table S1.

Additionally, dose reductions were documented in 28 patients. The specific adjustments for each patient are presented in Table S2. Most commonly, oxaliplatin (25/28 patients, 89.3%) and docetaxel (19/28, 67.9%) were reduced or omitted, while 5‐FU was dose‐reduced in 14 of 28 cases (50%), typically only as part of a general dose reduction. The frequency of cases with documented dose reductions was comparable between the groups (22.2% in the ‘FLOT incomplete’ group and 25.6% in the ‘FLOT complete’ group).

### 3.3 Kaplan–Meier analysis

#### 3.3.1 Total cohort

**FIGURE 2:**
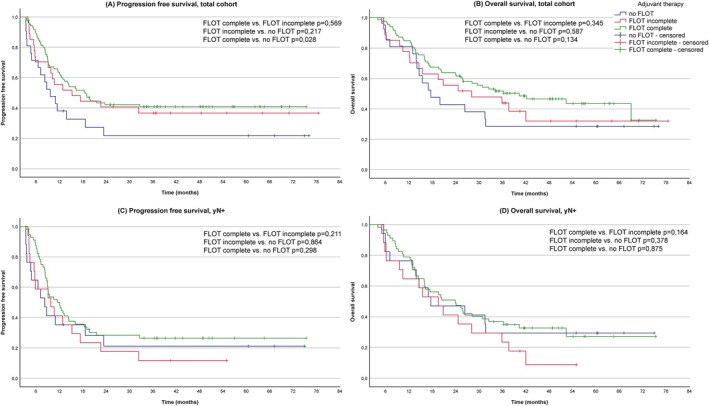
Kaplan–Meier survival curves for adjuvant therapy concepts: four cycles of FLOT/‘FLOT complete’ (green), one to three cycles of FLOT/‘FLOT incomplete’ (red), 0 cycles of FLOT/‘no FLOT’ (blue); (A) progression‐free survival (PFS) in the total cohort, (B) overall survival (OS) in the total cohort, (C) PFS in patients with lymph node metastasis (yN+), (D) OS in patients with lymph node metastasis (yN+); *p*‐values refer to pairwise log‐rank tests comparing the survival curves between each group. FLOT, a chemotherapy regimen consisting of docetaxel, oxaliplatin, leucovorin and 5‐fluorouracil.

Regarding PFS in the total cohort (Figure 2A), there is a statistically significant PFS advantage for patients who received four cycles of adjuvant FLOT (median PFS: 18.6 months, 95% confidence interval [CI]: 13.0–24.2 months) compared to patients with no adjuvant chemotherapy (median PFS: 9.6 months, 95% CI: 5.5–13.8 months) (*p* = .028, *p*‐value in this section refers to pairwise log‐rank test). The group of patients with one to three cycles of adjuvant FLOT shows similar PFS results to the ‘FLOT complete’ group (median PFS: 15.2 months, 95% CI: 3.9–26.6) (*p* = .569). Due to a smaller number of cases in this group and an early crossing of the survival curves, the difference compared to the ‘no FLOT’ group is visible, but not statistically significant (*p* = .217).

Concerning the OS of the total patient cohort (Figure 2B), there is a trend towards an improved survival in the ‘FLOT complete’ group (median OS: 40.3 months, 95% CI: 19.6–61.0) compared to the other groups, although the survival benefit is not statistically significant (*p* = .134 for comparison with ‘no FLOT’ and *p* = .345 for comparison with ‘FLOT incomplete’). The median OS for patients receiving one to three cycles is 28.1 months (95% CI: 3.7–52.5) and for patients without adjuvant therapy 17.6 months (95% CI: 10.9–24.4).

We also examined the reported effects in the overall cohort according to the regression grading system of Becker et al. within the subgroups with >50% viable tumour cells (TRG3/‘minor response’) and 10%–50% viable tumour cells (TRG2/‘partial response’). We did not observe any relevant difference in the effect of adjuvant FLOT therapy between patients with TRG2 and TRG3. The corresponding survival curves are provided in Figure S1.

#### 3.3.2 Patients with lymph node metastasis in surgical specimen (ypN+ cohort)

We additionally conducted a survival analysis for patients with lymph node metastasis in the surgical specimen (ypN+ cohort, *n* = 91). In this subgroup, the PFS advantage of the ‘FLOT complete’ group (median PFS: 12.1 months, 95% CI: 7.9–16.2) compared with the ‘no FLOT’ group (median PFS: 8.2 months, 95% CI: 5.3–11.2) is substantially reduced and no longer statistically significant (*p* = .298) (Figure 2C). Patients receiving one to three cycles of chemotherapy show comparable PFS data (median PFS 9.8 months, 95% CI: 3.3–16.3).

With regard to the OS data for patients in the ypN+ cohort, no statistically significant differences were observed between the three groups (Figure 2D). The median OS for patients receiving four cycles of adjuvant FLOT is 23.9 months (95% CI: 15.0–32.8), for patients with one to three cycles 19.5 months (95% CI: 11.2–27.4) and for patients without adjuvant therapy 17.6 months (95% CI: 1.8–33.5).

### 3.4 Univariate analysis and multivariate analysis

#### 3.4.1 Total cohort

In the univariate analysis (Table 2), completion of adjuvant FLOT therapy (‘FLOT complete’) was significantly associated with improved PFS (hazard ratio [HR] 0.540; 95% CI 0.307–0.950; *p* = .033). A similar trend was seen for OS, though statistical significance was not reached (HR 0.641; 95% CI 0.357–1.150; *p* = .136). Both higher ypT and ypN stages were significantly associated with worse prognosis. Specifically, advanced ypT stage (ypT3/4 vs. ypT1/2) predicted reduced PFS (HR 3.995; 95% CI 1.921–8.311; *p* < .001) and OS (HR 4.058; 95% CI 1.863–8.840; *p* < .001). Similarly, nodal involvement (ypN1–3 vs. ypN0) was associated with significantly shorter PFS (HR 3.892; 95% CI 2.182–6.943; *p* < .001) and OS (HR 3.810; 95% CI 2.090–7.210; *p* < .001).

**TABLE 2:** Univariate analysis.

	Progression‐free survival			Overall survival		
	HR	95% CI	*p*‐Value	HR	95% CI	*p*‐Value
Total cohort						
Sex (m vs. f)	0.771	0.418–1.422	.406	0.770	0.416–1.427	.407
Age (≤60 vs. >60)	1.490	0.964–2.303	.073	1.910	1.210–3.014	.005
No FLOT	Reference			Reference		
FLOT incomplete	0.633	0.319–1.254	.190	0.839	0.418–1.682	.620
FLOT complete	0.540	0.307–0.950	.033	0.641	0.357–1.150	.136
Type of surgery (oesophagectomy vs. gastrectomy)	0.902	0.567–1.434	.663	1.072	0.666–1‐725	.774
ypT (1/2 vs. 3/4)	3.995	1.921–8.311	<.001	4.058	1.863–8.840	<.001
ypN (0 vs. 1–3)	3.892	2.182–6.943	<.001	3.81	2.090–7.210	<.001
AEG I	Reference			Reference		
AEG II	0.808	0.482–1.354	.418	0.811	0.472–1.392	.447
AEG III	0.829	0.374–1.839	.645	1.247	0.576–2.698	.576
Stomach	0.663	0.354–1.242	.199	0.752	0.392–1.442	.390
Intestinal histology	Reference			Reference		
Diffuse histology	0.926	0.562–1.690	.926	1.388	0.803–2.399	.240
Mixed histology	1.696	1.028–2.798	.039	1.946	1.147–3.301	.014
Signet ring cells (no vs. yes)	0.678	0.350–1.314	.250	0.718	0.369–1.396	.329
yN+ cohort						
Sex (m vs. f)	0.941	0.481–1.841	.858	0.776	0.384–1.569	.481
Age (≤60 vs. >60)	1.312	0.813–2.115	.266	1.613	0.983–2.645	.058
No FLOT	Reference			Reference		
FLOT incomplete	1.050	0.499–2.208	.898	1.441	0.673–3.087	.347
FLOT complete	0.723	0.387–1.349	.308	0.953	0.498–1.824	.885
Type of surgery (oesophagectomy vs. gastrectomy)	1.066	0.643–1.770	.803	1.328	0.790–2.231	.284
ypT (1/2 vs. 3/4)	2.697	1.159–6.272	.021	2.553	1.100–5.926	.029
AEG I	Reference			Reference		
AEG II	0.665	0.374–1.184	.165	0.583	0.323–1.052	.073
AEG III	0.643	0.257–1.612	.347	0.978	0.410–2.328	.959
Stomach	0.783	0.392–1.564	.489	0.880	0.434–1.784	.722
Intestinal histology	Reference			Reference		
Diffuse histology	0.934	0.495–1.763	.834	1.407	0.754–2.627	.283
Mixed histology	1.649	0.966–2.813	.067	1.953	1.118–3.413	.019
Signet ring cells (no vs. yes)	0.621	0.297–1.301	.207	0.675	0.322–1.415	.299

Abbreviations: AEG, adenocarcinoma of the oesophagogastric junction, analogue Siewert‐classification; CI, confidence interval; f, female; a chemotherapy regimen consisting of docetaxel, oxaliplatin, leucovorin, and 5‐fluorouracil; HR, hazard ratio; m, male; ypN, pathologic nodal stage after neoadjuvant therapy; ypT, pathologic tumour stage after neoadjuvant therapy.

Multivariable analysis (Table 3) confirmed these results. Completion of adjuvant FLOT remained independently associated with improved PFS (HR 0.451; 95% CI 0.243–0.836; *p* = .011), though not with OS (HR 0.640; 95% CI 0.337–1.215; *p* = .172). ypT stage remained a strong independent predictor for both endpoints (PFS: HR 3.835; 95% CI 1.726–8.522; *p* < .001; OS: HR 3.288; 95% CI 1.398–7.736; *p* = .006), as did ypN status (PFS: HR 3.268; 95% CI 1.779–6.002; *p* < .001; OS: HR 3.637; 95% CI 1.853–7.140; *p* < .001).

**TABLE 3:** Multivariate analysis.

	Progression‐free survival			Overall survival		
	HR	95% CI	*p*‐Value	HR	95% CI	*p*‐Value
Total cohort						
Sex (m vs. f)	0.958	0.489–1.876	.900	0.846	0.430–1.667	.630
Age (≤60 vs. >60)	1.355	0.848–2.164	.204	1.718	1.058–2.790	.029
No FLOT	Reference			Reference		
FLOT incomplete	0.618	0.295–1.298	.204	0.958	0.461–1.990	.909
FLOT complete	0.451	0.243–0.836	.011	0.640	0.337–1.215	.172
Type of surgery (oesophagectomy vs. gastrectomy)	1.168	0.560–2.432	.679	1.523	0.694–3.340	.294
ypT (1/2 vs. 3/4)	3.835	1.726–8.522	<.001	3.288	1.398–7.736	.006
ypN (0 vs. 1–3)	3.268	1.779–6.002	<.001	3.637	1.853–7.140	<.001
AEG I	Reference			Reference		
AEG II	0.612	0.343–1.091	.096	0.589	0.323–1.074	.084
AEG III	0.619	0.257–1.491	.285	0.898	0.369–2.186	.813
Stomach	0.630	0.229–1.736	.372	0.545	0.188–1.581	.264
Intestinal histology	Reference			Reference		
Diffuse histology	1.071	0.546–2.102	.842	1.738	0.901–3.353	.099
Mixed histology	1.351	0.794–2.299	.268	1.618	0.933–2.807	.087
Signet ring cells (no vs. yes)	0.909	0.401–2.059	.819	0.737	0.317–1.716	.479
yN+ cohort						
Sex (m vs. f)	1.164	0.541–2.502	.697	0.977	0.442–2.160	.954
Age (≤60 vs. >60)	1.206	0.718–2.027	.479	1.517	0.895–2.573	.122
No FLOT	Reference			Reference		
FLOT incomplete	0.610	0.257–1.452	.264	0.996	0.436–2.273	.992
FLOT complete	0.405	0.192–0.855	.018	0.691	0.323–1.477	.340
Type of surgery (oesophagectomy vs. gastrectomy)	1.244	0.527–2.935	.618	1.598	0.662–3.860	.297
ypT (1/2 vs. 3/4)	3.379	1.299–8.791	.013	2.326	0.900–6.015	.082
AEG I	Reference			Reference		
AEG II	0.592	0.309–1.134	.114	0.533	0.277–1.027	.066
AEG III	0.676	0.245–1.867	.451	0.953	0.345–2.629	.925
Stomach	0.897	0.263–3.061	.862	0.650	0.183–2.304	.504
Intestinal histology	Reference			Reference		
Diffuse histology	0.817	0.349–1.916	.643	1.556	0.671–3.607	.303
Mixed histology	1.584	0.879–2.854	.126	1.939	1.062–3.542	.031
Signet ring cells (no vs. yes)	0.835	0.318–2.192	.715	0.609	0.223–1.665	.334

Abbreviations: AEG, adenocarcinoma of the oesophagogastric junction, analogue Siewert‐classification; CI, confidence interval; f, female; a chemotherapy regimen consisting of docetaxel, oxaliplatin, leucovorin, and 5‐fluorouracil; HR, hazard ratio; m, male; ypN, pathologic nodal stage after neoadjuvant therapy; ypT, pathologic tumour stage after neoadjuvant therapy.

#### 3.4.2 Patients with lymph node metastasis in surgical specimen (ypN+ cohort)

In the univariate analysis (Table 2), completion of adjuvant FLOT therapy (‘FLOT complete’) was not significantly associated with improved PFS (HR 0.723; 95% CI 0.387–1.349; *p* = .308) or OS (HR 0.953; 95% CI 0.498–1.824; *p* = .885). ypT stage (ypT3/4 vs. ypT1/2), on the other hand, was significantly associated with worse outcomes: PFS (HR 2.697; 95% CI 1.159–6.272; *p* = .021) and OS (HR 2.553; 95% CI 1.100–5.926; *p* = .029).

In the multivariate analysis (Table 3), FLOT complete emerged as a significant independent predictor for improved PFS (HR 0.405; 95% CI 0.192–0.855; *p* = .018), even though this effect was not evident in the univariate setting. For OS, no significant benefit was observed (HR 0.691; 95% CI 0.323–1.477; *p* = .340). ypT stage remained an independent risk factor for both endpoints, significantly for PFS (HR 3.379; 95% CI 1.299–8.791; *p* = .013) and with a nonsignificant trend for OS (HR 2.326; 95% CI 0.900–6.015; *p* = .082).

### 3.5 IPTW‐weighted analysis

Following multivariable analysis, an IPTW‐weighted analysis was performed to further reduce confounding. We provide its results in Figure S2. Kaplan–Meier curves are broadly consistent with the unweighted analysis, reflecting a balanced distribution of key baseline characteristics across treatment groups, as shown in the patients' characteristics table. A slightly clearer dose‐dependent effect was seen in the total cohort, and in the ypN+ cohort, the PFS benefit of FLOT‐complete was confirmed, though less pronounced than in the total cohort. The strong statistical significance primarily confirms true group differences and also reflects increased estimate precision due to the larger effective sample size introduced by weighting (*n* = 306 for total cohort and *n* = 213 for ypN+ cohort).

## 4 DISCUSSION

To the best of our knowledge, this is one of the first studies to investigate the effect of adjuvant FLOT‐therapy in patients with low tumour regression in OGA. For 2022, the European Cancer Information System (ECIS) estimates the incidence of gastric and oesophageal cancer at approximately 105,000 patients/year.^15^ Prospective studies report that 56%–58% of patients experience low tumour regression (>10% viable tumour cells) after neoadjuvant FLOT, making the question of continuation of adjuvant therapy relevant for a significant number of patients.^9^, ^16^, ^17^

In our study, two key findings emerged: (1) Contrary to our initial hypothesis, full adjuvant therapy with FLOT (four cycles) was superior to active surveillance (0 cycles) in terms of PFS across the overall cohort. (2) However, in patients with lymph node metastases in surgical specimens (yN+ cohort), this effect was drastically reduced due to the generally worse outcome of this subgroup and no OS difference was observed for these patients. Based on these (retrospective) findings, continuation of perioperative FLOT therapy should be recommended even for patients with low tumour regression. However, this approach should be critically discussed for patients in the yN+ cohort, given the reduced benefit observed in this subgroup. The role of incomplete adjuvant therapy (one to three cycles) remains unclear, though its effect in the total patient cohort likely lies between that of the other two subgroups. With regard to OS, patient‐related factors such as age, comorbidities or postoperative recovery may have influenced outcomes in the ‘FLOT incomplete’ and ‘no FLOT’ groups, although there was no clear indication of differences in ECOG performance status. These factors are less likely to affect PFS, which is more directly related to tumour recurrence. Notably, the IPTW‐weighted analysis, which adjusts for confounding variables, supports a dose‐ and therefore rather treatment‐related effect of adjuvant FLOT in the overall cohort.

The role of adjuvant therapy in OGA patients, independent of regression grade, has been examined in various studies with inconsistent results.^18^, ^19^, ^20^, ^21^ In one of the largest cohorts, Sisic et al. (299 OGA patients) found no survival benefit from adjuvant chemotherapy across different perioperative regimens.^18^ However, in the subgroup receiving perioperative FLOT (*n* = 148), they observed a benefit from continuing the adjuvant phase of FLOT‐therapy. Similarly, a recent study by Li et al. (174 cT3‐4N+ OGA patients) demonstrated that adjuvant therapy improves OS compared to neoadjuvant chemotherapy plus surgery alone, with a survival benefit also seen in ypN+ patients.^19^ In contrast, Ballhausen et al. (124 OGA patients) showed that adjuvant chemotherapy provides a survival benefit only in ypN0 patients, with no effect in those with lymph node metastasis in surgical specimen, which aligns with our findings.^20^ Furthermore, the above‐mentioned studies only include few subgroup analyses that explicitly focus on patients with low tumour regression.^18^, ^19^ However, these studies suggest that such patients may also benefit from adjuvant therapy, although the results cannot be directly related to ours due to the inclusion of various perioperative chemotherapy regimens.

An important point of reference in this context is the recently published SPACE‐FLOT trial, which explicitly investigated the impact of adjuvant FLOT therapy in relation to histopathological tumour regression. Their results are fully in line with our findings, showing a survival benefit from adjuvant FLOT in patients classified as ‘partial responders’. While the large cohort lends robustness to their conclusions, it is important to note that tumour regression was assessed using seven different TRG systems. As discussed in the introduction, the definition of ‘partial response’ encompasses a much broader range of tumour regression than the Becker classification, potentially including patients with 1%–99% viable tumour cells. Therefore, no conclusion should be drawn from the SPACE‐FLOT results suggesting that patients classified as minor responders according to Becker (i.e., TRG3) should not receive adjuvant therapy, as they may well have been included in the ‘partial response’ category and contributed to the observed benefit. The cohort we analyzed (patients with 10–100% viable tumour cells) largely overlaps with the SPACE‐FLOT ‘partial response’ group and yielded comparable results. Subgroup analyses, particularly ypN+ patients, were not reported in SPACE‐FLOT—making our data a relevant and complementary contribution for this group of patients.

To date, the mechanisms driving resistance to FLOT in OGA patients remain unclear.^22^ The largest effort to identify genetic predictors of response to systemic therapies in oesophageal and gastric cancer by Janjigian et al. (prospective next‐generation sequencing of 187 HER2‐negative OGA patients) showed that no single mutant allele or gene was significantly associated with treatment response.^23^ Furthermore, microsatellite‐instable (MSI‐H) tumours, which seem to have intrinsic resistance to chemotherapy, account for less than 10% of OGA cases.^9^, ^10^ New approaches to predicting therapy response are therefore needed. Recent studies on circulating tumour DNA (ctDNA) show promising results and may enable response‐guided therapy approaches in the future.^24^, ^25^ Nevertheless, these approaches have not yet been clinically established for OGA, meaning therapy decisions still rely on clinicopathological information. Tumour regression remains a potential indicator of chemotherapy response in current practice; although, as shown in this study, low regression in the surgical specimen is not necessarily a reliable marker for treatment efficacy.

This study has some limitations. First and foremost, it is a retrospective analysis, which means the nature of this study remains hypothesis‐generating. While the 134 patients represent the largest single‐centre cohort studied with low tumour regression in OGA to date, larger sample sizes from multicentre studies like the SPACE‐FLOT cohort and prospective data are necessary to confirm our findings. Single‐centre studies inherently carry a risk of hidden bias, for example related to comorbidities, postoperative complications or patient compliance. At the same time, the single‐centre design also offers advantages, such as uniform surgical procedures, standardised pathological assessment (particularly including TRG grading), and consistent follow‐up procedures. Furthermore, there is ambiguity due to reported dose reductions in patients who received adjuvant therapy; this concerns 28 out of 113 (24.8%) patients undergoing adjuvant treatment (22.2% for ‘FLOT incomplete’ and 25.6% for ‘FLOT complete’). However, we cannot definitively exclude unreported dose reductions, which must be taken into account during the evaluation.

Given the significant number of patients affected by the question of adjuvant therapy continuation, it is surprising that there is limited data on the efficacy of adjuvant treatment. This may partly be due to inadequate follow‐up strategies in patient care, arising from the involvement of various treating specialties (surgical treatment in hospital and chemotherapy treatment in outpatient oncology clinics). In our patient cohort, follow‐up data could not be obtained for 30 patients (17.6%). Conducting structured follow‐up and data collection for these patients must be prioritised to facilitate hypothesis‐generating research. This could lead to an optimisation of treatment for OGA patients in the future.

Taken together, the adjuvant continuation of FLOT is superior to active surveillance in patients with low regression and OGA, although the effect is drastically reduced and clinically non‐significant in patients with lymph node metastases in the surgical specimen. Prospective studies, such as those focusing on ypN+ patients with low tumour regression, could aid in individualising treatment strategies and identifying patients who may benefit less from adjuvant FLOT therapy in OGA.

## AUTHOR CONTRIBUTIONS

**Max Kraemer:** Conceptualization; methodology; formal analysis; investigation; data curation; writing – original draft; writing – review and editing. **Naita M. Wirsik:** Writing – review and editing; resources. **Hakan Alakus:** Resources; writing – review and editing. **Hans A. Schloesser:** Resources; writing – review and editing. **Hans Fuchs:** Resources; writing – review and editing. **Wolfgang Schroeder:** Resources; writing – review and editing. **Christiane J. Bruns:** Resources; writing – review and editing. **Su Ir Lyu:** Formal analysis; data curation; writing – review and editing. **Friederike Baehr:** Investigation; writing – review and editing. **Thomas Zander:** Conceptualization; methodology; data curation; writing – original draft; writing – review and editing. **Alexander Quaas:** Conceptualization; methodology; data curation; writing – original draft; writing – review and editing.

## CONFLICT OF INTEREST STATEMENT

The authors declare that they have no conflicts of interest related to this work, except for Hans Fuchs, who reports relationships with Medtronic (Advisory Board), Stryker (Consultant) and Distalmotion (Consultant).

## ETHICS STATEMENT

This retrospective study was performed according to the criteria of the ethics committee of the University Hospital of Cologne and according to the Helsinki Declaration (Ethics Committee of the Medical Faculty of University of Cologne: registration no. 13‐091). Informed consent was obtained from all patients in the study concerning the use of tumour material for research activities, data exchange and the use of follow‐up data in collaboration with outpatient clinics.

## Supporting information


**Data S1.** Supporting Information.

## Data Availability

The data that support the findings of our study are available on request from the corresponding author.
