# Supplementary material for: Adjuvant FLOT provides survival benefit for oesophagogastric junction and gastric adenocarcinoma patients with low tumour regression after neoadjuvant chemotherapy
Source: Int J Cancer. 2025 Jul 22;157(12):2558–68. doi: 10.1002/ijc.70048 (PMC12541569; doi:10.1002/ijc.70048)
Supplement: Supplementary file 1 — Data S1. Supporting Information. [file IJC-157-2558-s001.pdf]

Adjuvant FLOT provides survival benefit for esophagogastric junction and gastric adenocarcinoma patients with low tumor regression after neoadjuvant chemotherapy

Max Kraemer, Naita M Wirsik, Hakan Alakus, Hans A Schloesser, Hans Fuchs, Wolfgang Schroeder, Christiane J Bruns, Su Ir Lyu, Friederike Baehr, Thomas Zander and Alexander Quaas

Table of contents

Supplementary table 1……………………………………………………………………2

Supplementary table 2……………………………………………………………………2

Supplementary table 3……………………………………………………………………3

Supplementary figure 1……………………………………………………………………4

Supplementary figure 2……………………………………………………………………5

Supplementary table 1 Reasons for no adjuvant FLOT-therapy

Reason	Patients
Performance status	42.9% (N=9)
Delayed postoperative recovery	23.8% (N=5)
Patient refusal	9.5% (N=2)
Clinical decision	9.5% (N=2)
Unknown	9.5% (N=2)
Drug toxicity from neoadjuvant therapy	4.8% (N=1)

Supplementary table 2 Reported dose reductions: The dose reductions refer to the number of administered cycles. (For example, “case 73” received 4 treatment cycles: 1 cycle at 100% dose and 3 cycles at 80% of all agents, resulting in an average of 85% of the maximum possible cumulative dose over 4 cycles)

Case	Cycles	5-FU	Platine	Taxane	Description
6	4	25%	100%	100%	from 2nd cycle without 5-FU
9	4	100%	87.50%	100%	from 3rd cycle dose reduction platine to 75%
12	4	93.75%	93.75%	93.75%	4th cycle in 75% of all agents
33	4	100%	50%	100%	from 3rd cycle without platine
34	1	75%	50%	75%	1 cycle with dose reduction of platine to 50%
37	4	100%	100%	0%	no taxane adjuvant
40	4	75%	75%	75%	all cycles in 75%
45	4	100%	68.75%	75%	all cycles with dose reduction of platine and taxane to 75%, 1 cycle of platine in 50%
55	3	100%	66.67%	100%	3 cycles, 3rd cycle without platine
63	3	66.67%	66.67%	0%	cycle 1 and 2 dose reduction of 5-FU+platine to 80%, cycle 3 dose reduction of 5-FU+platine to 60%, no taxane
66	4	100%	50%	100%	all cycles with dose reduction of platine to 50%
73	4	85%	85%	85%	from 2nd cycle dose reduction of all agents to 80%
82	4	100%	66.67%	87.5%	2nd cycle with dose reduction of platine and taxane to 50%, 3rd and 4th cycle with dose reduction of platine to 75%
88	4	75%	75%	75%	all cycles with dose reduction of all agents to 75%
94	3	100%	41.67%	100%	3 cycles, 1st with dose reduction of platine to 75%, 2nd with dose redution of platine to 50% and 3rd without platine
95	4	100%	25%	25%	2nd4th cycle without platine and taxane
100	4	93.75%	93.75%	93.75%	4th cycle in 75% of all agents
101	4	62.50%	43.75%	62.50%	1st cycle all agents in 75%, 2nd cycle 5-FU+taxane in 75%, platine in 50%, 3rd cycle all agents in 50%, 4th cycle 5-FU+taxane in 50%, no platine
102	4	100%	25%	25%	from 2nd cycle without platine and taxane
103	3	100%	0%	100%	3 cycles, no platine
109	4	87.50%	87.50%	87.50%	4th cycle in 50% of all agents
110	4	93.75%	93.75%	93.75%	4th cycle in 75% of all agents
113	4	100%	100%	0%	no taxane adjuvant

114	4	100%	47.50%	100%	2nd cycle with dose reduction of platine to 90%, 3rd and 4th cycle without platine
116	4	75%	75%	50%	all cycles with dose reduction to 75%, last cycle with dose reduction of taxane to 50%
125	1	50%	50%	50%	1 cycle with dose reduction to 50%
126	4	80%	80%	80%	all cycles with dose reduction to 80%
129	4	100%	50%	100%	3rd and 4th cycle with dose reduction of platine to 50%

Supplementary Table 3 Biomarker

	**All**	**FLOT complete (4 cycles)**	**FLOT incomplete (1-3 cycles)**	**No FLOT (0 cycles)**	**p-value**
HER2 amplification					
yes	3 (2.2%)	2 (2.3%)	1 (3.7%)	0 (0.0%)	0.828
no	40 (29.9%)	26 (30.2%)	10 (37.0%)	4 (19.0%)	
n.a.	91 (67.9%)	58 (67.4%)	16 (59.3%)	17 (81.0%)	
PD-L1, CPS>5					
yes	4 (3.0%)	4 (4.7%)	0 (0.0%)	0 (0.0%)	0.237
no	7 (5.2%)	5 (5.8%)	2 (7.4%)	0 (0.0%)	
n.a.	123 (91.8%)	77 (89.5%)	25 (92.6%)	21 (100%)	
MSI-h					
yes	1 (0.7%)	1 (1.2%)	0 (0.0%)	0 (0.0%)	0.862
no	39 (29.1%)	30 (34.9%)	7 (25.9%)	2 (9.5%)	
n.a.	94 (70.1%)	55 (64.0%)	20 (74.1%)	19 (90.5%)	

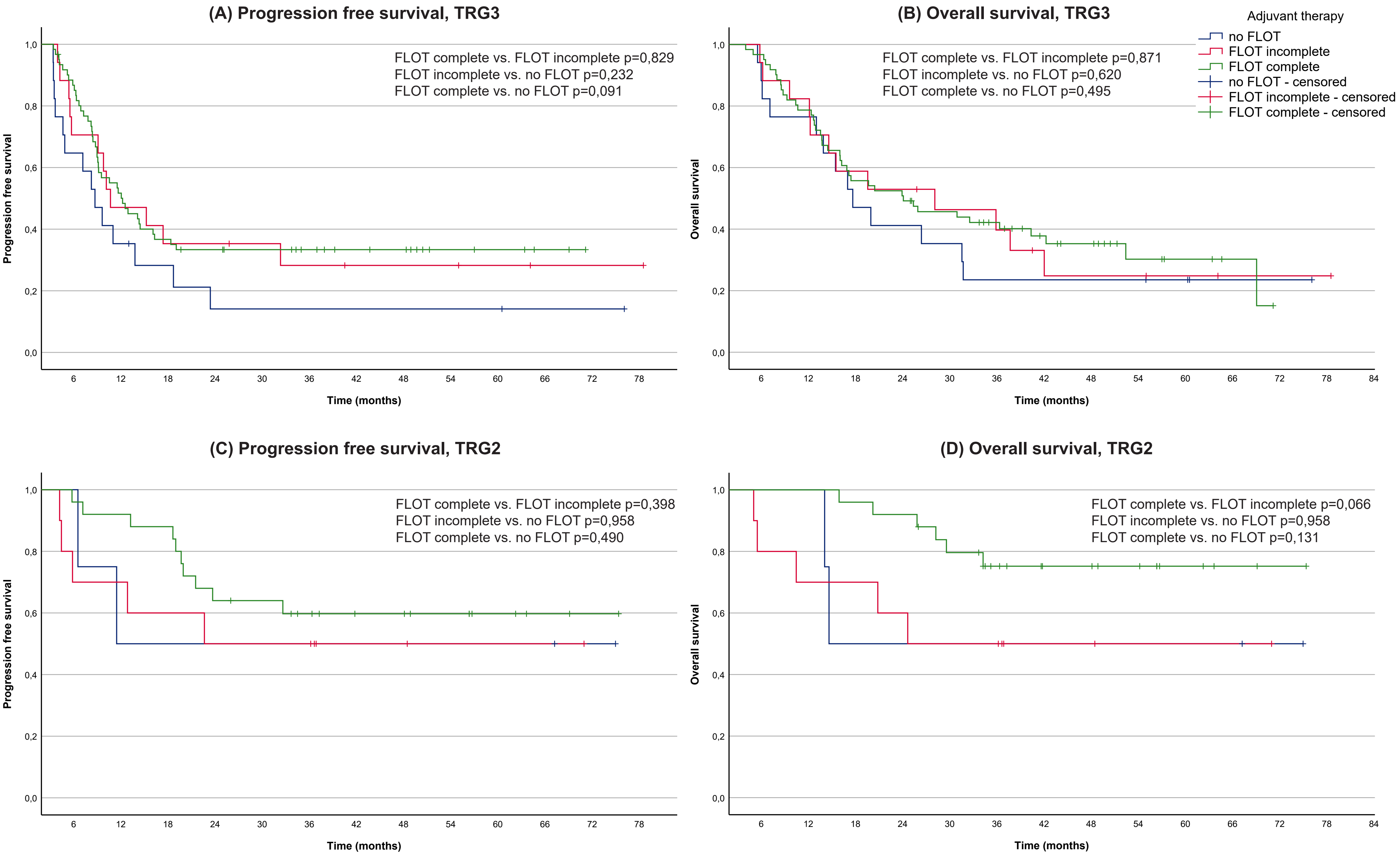

Supplementary figure 1: Kaplan-Meier survival curves for adjuvant therapy concepts: 4 cycles of FLOT/‘FLOT complete‘ (green), 1-3 cycles of FLOT/‘FLOT incomplete‘ (red), 0 cycles of FLOT/‘no FLOT‘ (blue); (A) PFS in patients with > 50% viable tumor cells in surgical specimen (TRG3), (B) OS in patients with > 50% viable tumor cells in surgical specimen, (C) PFS in patients with 10-50% viable tumor cells in surgical specimen (TRG2), (D) OS in patients with 10-50% viable tumor cells in surgical specimen (TRG2) p-values refer to pairwise log-rank tests comparing the survival curves between each group

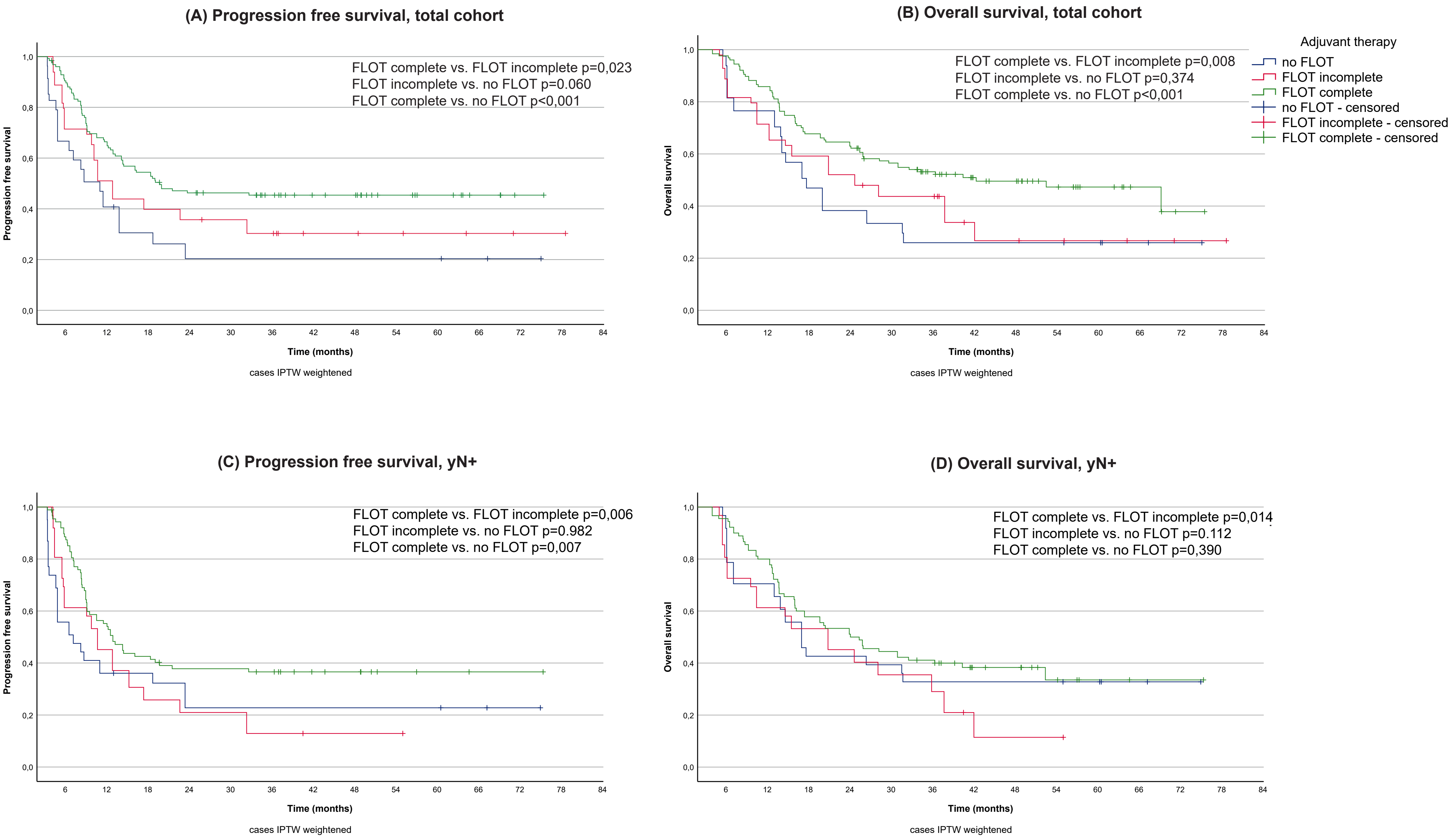

Supplementary Fig. 2 IPTW weightened Kaplan-Meier survival curves for adjuvant therapy concepts: 4 cycles of FLOT/'FLOT complete' (green), 1-3 cycles of FLOT/'FLOT incomplete' (red), 0 cycles of FLOT/'no FLOT' (blue); (A) PFS in the total cohort, (B) OS in the total cohort, (C) PFS in patients with lymph node metastasis (yN+), (D) OS in patients with lymph node metastasis (yN+) p-values refer to pairwise log-rank tests comparing the survival curves between each group
